# Cost-Effectiveness of Virtual Reality Cognitive Behavioral Therapy for Psychosis: Health-Economic Evaluation Within a Randomized Controlled Trial

**DOI:** 10.2196/17098

**Published:** 2020-05-05

**Authors:** Roos Pot-Kolder, Wim Veling, Chris Geraets, Joran Lokkerbol, Filip Smit, Alyssa Jongeneel, Helga Ising, Mark van der Gaag

**Affiliations:** 1 Department of Clinical, Neuro and Developmental Psychology Vrije Universiteit Amsterdam Netherlands; 2 Department of Psychiatry University Medical Centre Groningen University of Groningen Groningen Netherlands; 3 Centre of Economic Evaluation and Machine Learning Trimbos Institute Utrecht Netherlands; 4 Department of Health Care Policy Harvard Medical School Boston, MA United States; 5 Department of Epidemiology and Biostatistics University Medical Centers Amsterdam Amsterdam Netherlands; 6 Department of Psychosis Research Parnassia Psychiatric Institute The Hague Netherlands

**Keywords:** psychosis, virtual reality, cognitive behavioral therapy, cost-effectiveness

## Abstract

**Background:**

Evidence was found for the effectiveness of virtual reality-based cognitive behavioral therapy (VR-CBT) for treating paranoia in psychosis, but health-economic evaluations are lacking.

**Objective:**

This study aimed to determine the short-term cost-effectiveness of VR-CBT.

**Methods:**

The health-economic evaluation was embedded in a randomized controlled trial evaluating VR-CBT in 116 patients with a psychotic disorder suffering from paranoid ideation. The control group (n=58) received treatment as usual (TAU) for psychotic disorders in accordance with the clinical guidelines. The experimental group (n=58) received TAU complemented with add-on VR-CBT to reduce paranoid ideation and social avoidance. Data were collected at baseline and at 3 and 6 months postbaseline. Treatment response was defined as a pre-post improvement of symptoms of at least 20% in social participation measures. Change in quality-adjusted life years (QALYs) was estimated by using Sanderson et al’s conversion factor to map a change in the standardized mean difference of Green’s Paranoid Thoughts Scale score on a corresponding change in utility. The incremental cost-effectiveness ratios were calculated using 5000 bootstraps of seemingly unrelated regression equations of costs and effects. The cost-effectiveness acceptability curves were graphed for the costs per treatment responder gained and per QALY gained.

**Results:**

The average mean incremental costs for a treatment responder on social participation ranged between €8079 and €19,525, with 90.74%-99.74% showing improvement. The average incremental cost per QALY was €48,868 over the 6 months of follow-up, with 99.98% showing improved QALYs. Sensitivity analyses show costs to be lower when relevant baseline differences were included in the analysis. Average costs per treatment responder now ranged between €6800 and €16,597, while the average cost per QALY gained was €42,030.

**Conclusions:**

This study demonstrates that offering VR-CBT to patients with paranoid delusions is an economically viable approach toward improving patients’ health in a cost-effective manner. Long-term effects need further research.

**Trial Registration:**

International Standard Randomised Controlled Trial Number (ISRCTN) 12929657; http://www.isrctn.com/ISRCTN12929657

## Introduction

Psychotic disorders impose a large disease burden—morbidity plus mortality—on the population, and in its wake, substantial economic costs occur for society and health care systems. The main drivers of societal costs of schizophrenia are health care costs and productivity losses, but patients and their families also incur substantial costs [1]. Low participation rates of individuals with psychosis in the labor market are an important cause of productivity losses, while the main contributor to health care costs are in-patient psychiatric admissions [2]. All in all, treatment costs of psychotic disorders consume a significant part of health care budgets in European countries [3].

Paranoid ideation is a common delusion in individuals with a psychotic disorder. Even when medicinal treatment is successful, paranoid thoughts and anxiety often remain because of conditioned avoidance and other acquired safety behaviors in social situations [4]. Social avoidance hinders recovery in social participation for patients and keeps unemployment rates as high as 70%-85% [5,6]. A poor social network contributes to stigma and a lack of empowerment, resulting in more depressive symptoms and lower quality of life [7]. A smaller social network size is associated with more severe overall psychiatric and negative symptoms [8]. Virtual reality-based cognitive behavioral therapy (VR-CBT) was found to be an effective treatment for paranoid ideation in individuals with a psychotic disorder [9,10]. The use of virtual reality (VR) treatment in clinical practice is expected to become more widespread as VR technology becomes more readily available [11]. Therefore, information on the cost-effectiveness of this kind of treatment is required. This study was designed to evaluate whether adding VR-CBT to treatment as usual (TAU) would be effective in treating paranoid ideation in a cost-effective way with respect to improving social participation. A trial-based cost-effectiveness analysis (CEA) was conducted using data collected in seven outpatient mental health care services in the Netherlands, comparing add-on VR-CBT with TAU alone. This paper aims to determine the short-term (ie, 6-month) cost-effectiveness of VR-CBT from a societal perspective.

## Methods

### Research Design

The health-economic evaluation was embedded in a randomized controlled trial evaluating VR-CBT in 116 patients with a psychotic disorder suffering from paranoid ideation [10]. The VR-CBT study was a randomized, controlled, single-blind multicenter trial in two parallel groups, comparing add-on VR-CBT to TAU alone over a period of 6 months [10]. This study was approved by the Vrije Universiteit (VU University) Medical Ethics Committee for mental health service research and was registered retrospectively at the ISRCTN (International Standard Randomised Controlled Trial Number) registry (ISRCTN12929657). The trial protocol is provided elsewhere [12]. Four virtual social environments—a street, bus, café, and supermarket—were created with Vizard software (WorldViz). Within the environment, participants could move by operating a Logitech F310 Gamepad. They used a Sony HMZ-T1/T2/T3 head-mounted display with a high-definition resolution of 1280 × 720 per eye, a 51.6 diagonal field of view, and a 3DOF (3 degrees of freedom) tracker for head rotation. VR-CBT therapists were psychologists with at least basic cognitive behavioral therapy (CBT) training. They received 2 days of training in VR-CBT. The VR-CBT manual described a structured treatment plan for all 16 sessions. Therapists were supervised in a group for 4 hours every month by two VR-CBT specialists.

### Recruitment

Participants were recruited at seven treatment centers in the Netherlands between April 1, 2014, and December 31, 2015. To be included, participants had to meet the following criteria: (1) 18-65 years of age; (2) DSM-IV (Diagnostic and Statistical Manual of Mental Disorders, Fourth Edition) diagnosis of schizophrenia, schizophreniform disorder, schizoaffective disorder, delusional disorder, or psychotic disorder not otherwise specified; (3) suffering from at least mild paranoia, as assessed by Green’s Paranoid Thoughts Scale (GPTS) (score of >40); and (4) self-report of avoiding at least one social situation. Exclusion criteria were as follows: (1) insufficient competency of Dutch language; (2) IQ below 70; and (3) a concurrent diagnosis of epilepsy. Assessments were performed at baseline and at 3 and 6 months postbaseline.

### Interventions

All participants continued to receive TAU (ie, antipsychotic medication, regular contact with a psychiatrist to manage symptoms, and regular contact with a psychiatric nurse). Participants in the experimental condition also received therapist-led VR-CBT. VR-CBT treatment consisted of 16 biweekly sessions of 60 minutes each, using 40 minutes for exposure and behavioral exercises in virtual social environments. The therapist used an individual case formulation to help patients falsify their harm expectancies. No homework exercises were given between VR-CBT sessions. The treatment protocol, in Dutch, is available from the corresponding author.

### Outcome Measures

#### Overview

We conducted both a CEA with three measures of improved social participation as outcome and a cost-utility analysis (CUA) with quality-adjusted life years (QALYs) gained as outcome. The outcome measures are described in more detail below.

#### Social Participation

The outcome of interest in the CEA was social participation. Social participation was operationalized in three ways: (1) objective social participation as the amount of time spent with others, (2) subjective social participation as momentary anxiety, and (3) subjective social participation as momentary paranoia. *Momentary* in this context meant that it was measured in real life during social company. All three outcomes were assessed in real time using the ecological sampling method (ESM). ESM is a structured diary method in which individuals are asked in daily life to report their thoughts, feelings, and symptoms, as well as the appraisal of the present social context. To that end, all participants carried an electronic device (PsyMate) for the ESM assessments. The device beeped at semirandom moments 10 times a day over 6 days. At each beep, the device collected self-assessments on a 7-point Likert scale ranging from 1 (not at all) to 7 (very). A positive treatment response on each of the three outcome measures was defined as an improvement of at least 20% at 6 months follow-up relative to the patient’s baseline score.

#### Quality-Adjusted Life Year

The outcome in the CUA was the QALY derived from the GPTS [13]. The GPTS is an established broad measure of paranoid-delusional functioning that has long been used as an outcome measure. This instrument was chosen to be able to compare results with earlier CUA research on the subject. Mean GPTS scores at each measurement were first converted into the standard mean difference (SMD) by dividing the raw mean change scores by the SD of the GPTS at baseline in the control condition. In a next step, we used Sanderson et al’s conversion factor [14] of 0.1835 (ie, the average of 0.209 using a rating scale and 0.158 using time trade-off), such that a change of 1 standard unit (ie, SMD) on the GPTS is equal to a corresponding change of 0.18 utility. The utility is a quality of life valuation and is needed to compute QALY gains in the VR-CBT condition relative to the TAU condition over the full 6 months between baseline and follow-up.

#### Resource Use and Costing

Societal costs were computed by adding (1) the direct medical costs of health care services use including the costs of antipsychotic medication and, in the experimental condition, the additional costs of adjunctive VR-CBT treatment; (2) direct nonmedical costs of travel; and (3) indirect costs stemming from lower productivity. For each participant, cost data over the last 3 months were collected at each of three measurement points. Resource use data, for costing, were collected using the Trimbos Institute and Institute of Medical Technology Assessment Questionnaire for Costs Associated with Psychiatric Illness (TiC-P) [15]. The TiC-P is the most widely used health service interview in the Netherlands. It consists of questions on the number of contacts by type of health care provider and questions on productivity losses. A health service questionnaire is a valid and reliable method for quantifying costs in trial-based economic evaluations in health care [16]. A cross-validation sample comparing TiC-P self-report to electronic patient files showed all data to be reliable, except for the number of reported sessions with a psychologist (data available upon request from first author). Not all patients had understood that they needed to incorporate the 16 VR-CBT sessions into their TiC-P self-report. This information was, therefore, 100% cross-checked using electronic patient files. The main cost driver was admission to psychiatric hospitals, so the number of days admitted to a psychiatric hospital was also 100% cross-checked against electronic patient files and corrected where needed.

Direct medical costs were calculated by multiplying health service units (eg, sessions, visits, and hospital days) with their standard economic cost price (see Multimedia Appendix 1). We also added the medication costs, consisting of antipsychotic and antidepressant medication. Corresponding costs were calculated as the cost price per standard daily dose, as reported in the Dutch Pharmaceutical Compass [17], multiplied by the number of prescription days, plus the pharmacist’s dispensing costs of €6 per monthly prescription or €12 for a first-time prescription [18].

#### Virtual Reality Costs

For VR therapy hardware, software and training costs were calculated. Total yearly costs for one VR system was €23,995, according to CleVR BV, a company who builds VR sets. Yearly costs for training and supervision of the psychologists was €13,400. Per-patient costs per 16 VR-CBT treatment sessions was €373.95.

#### Travel Costs

Travel costs arose when participants had to make return trips for receiving health care at health services. Travel costs were computed as the average distance to a health service (7 km) multiplied by the costs per km (€0.21) [18].

#### Productivity Costs

Research assistants monitored changes in the participants’ work status at baseline and at 3 and 6 months postbaseline using the TiC-P. Productivity losses in paid work were calculated according to the human capital approach [19], reflecting changes in the contractual number of hours worked per week and adjusting these for work-loss days arising from sick leave over the full period of 6 months using gender-specific hourly productivity costs [18]. Costs were originally expressed in Euros for the reference year 2014, but indexed to 2015 using the consumer price index as reported by Statistics Netherlands. In the reference year 2015, 1 Euro in the Netherlands equaled 1.235 US$.

### Statistical Analysis

#### Imputation

Following the CONSORT (Consolidated Standards of Reporting Trials) and CHEERS (Consolidated Health Economic Evaluation Reporting Standards) guidelines, all our analyses adhered to the intention-to-treat principle. To that end, missing values were imputed using multiply imputed chained equations (MICE) for nonparametric data with M of 100 bootstraps for each incomplete variable. Baseline variables predictive of effects (ie, QALYs and treatment response) were used for imputation, such as baseline data of the variable with missing values, treatment condition, ethnicity, education, sex, age, and safety behaviors at baseline. Safety behaviors, such as avoiding eye contact or escaping from social situations, were measured using the Safety Behaviour Questionnaire-Persecutory Delusions (SBQ-PD) [20]. *Time spent with others* showed a large difference at baseline despite randomization and was added as covariate in the CEA where *time spent with others* was used as the treatment response outcome of interest.

#### Main Analysis

Both the CUA and CEA were conducted from the societal perspective. In each of these analyses, the incremental cost-effectiveness ratios (ICERs) were calculated as the between-group cost difference divided by the between-group effect difference. The ICER is interpreted as the additional costs per additional unit effect (ie, per additional treatment responder; per QALY gained). Cost and effect differences were obtained from seemingly unrelated regression equations of costs and effects, thus allowing for correlated residuals in the equations. The seemingly unrelated regression equations (SURE) models were bootstrapped 5000 times. In each bootstrap step, the mean cost differences and the mean outcome differences were computed and these were plotted on the cost-effectiveness plane. Finally, cost-effectiveness acceptability curves (CEACs) were graphed. CEACs inform decision makers about the likelihood that an intervention is deemed cost-effective, given a range of willingness-to-pay ceilings for gaining 1 QALY and gaining 1 treatment responder. All analyses were conducted in Stata, version 13.1 (StataCorp).

#### Sensitivity Analyses

The following sensitivity analyses were carried out. First, a sensitivity analysis was done including safety behavior at baseline as a covariate because despite randomization there was a significant difference at baseline, and it was found to be the main mediator in reducing paranoid ideation [10]. Second, a sensitivity analysis was done including psychiatric admission costs at baseline as a covariate because there was a large difference between groups at baseline. Third, a sensitivity analysis was done including both safety behavior at baseline and psychiatric admission costs at baseline as covariates in the model.

## Results

### Overview

**Figure 1:**
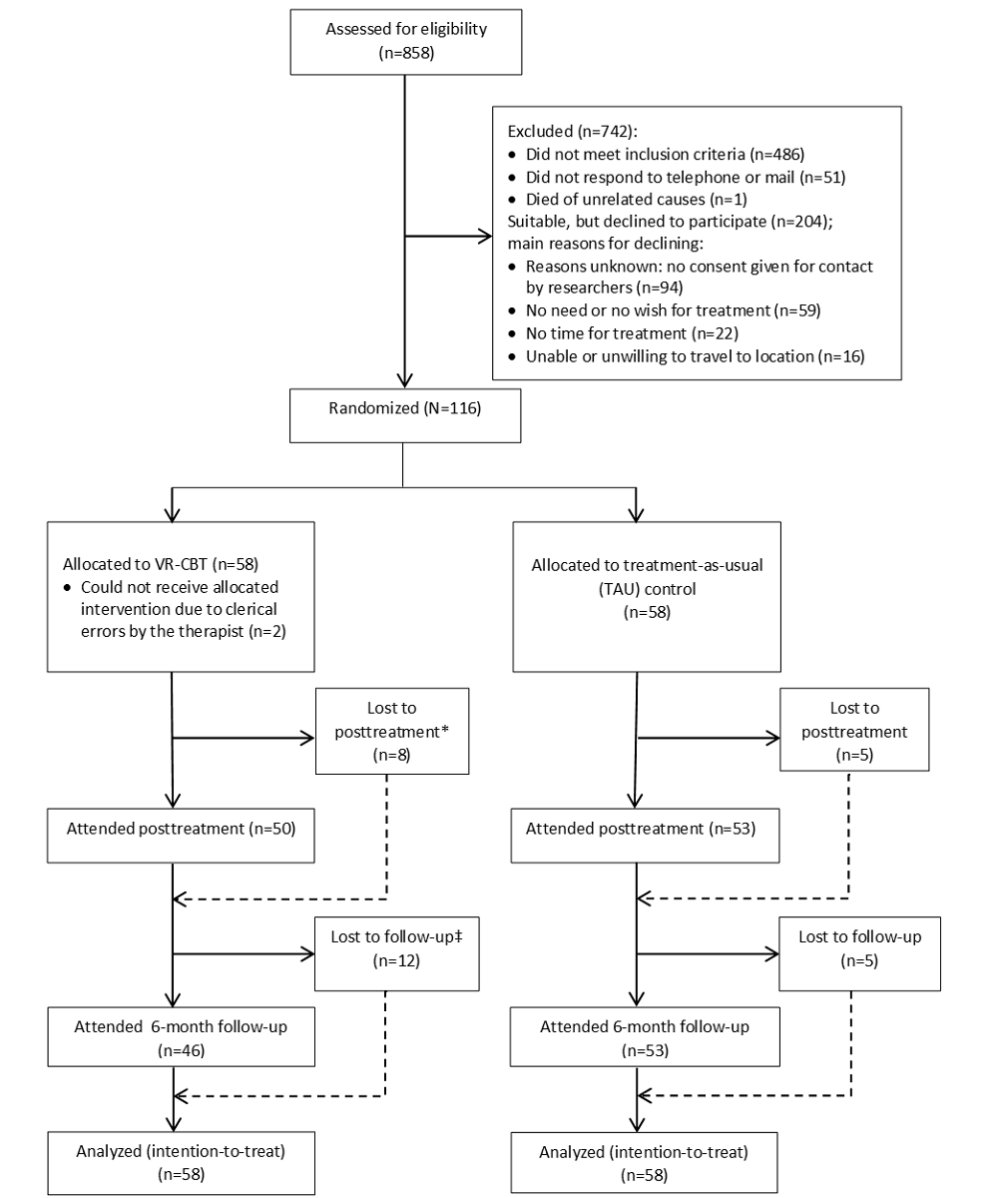
Trial flow diagram. *Specification of participants lost to posttreatment: 6 declined further participation and 2 were lost due to clerical errors by therapist. ‡Specification of participants lost to follow-up: 9 declined further participation, 1 died of unrelated causes, and 2 were lost due to clerical errors by therapist. First published in Lancet Psychiatry (Pot-Kolder et al, 2018). VR-CBT: virtual reality-based cognitive behavioral therapy.

After providing informed consent, 116 participants agreed to participate: 58 (50.0%) in the control condition and 58 (50.0%) in the experimental condition (see Figure 1).

Baseline characteristics of the sample can be found in Table 1. Results of costs and outcomes can be found in Table 2. A small group of participants was responsible for a large portion of the baseline costs, largely related to hospital admissions. The total days of psychiatric admissions were 233 days at baseline, 101 days posttreatment, and zero days at follow-up for the VR-CBT group. The total days of psychiatric admissions were 138 days at baseline, 20 days posttreatment, and 68 days at follow-up for the TAU group.

**Table 1:** Characteristics of the study sample at baseline.

Characteristic		VR-CBT^a^ (n=58)	Treatment as usual (TAU) (n=58)
Gender (male), n (%)		40 (69)	42 (72)
Age in years, mean (SD)		36.5 (9.8)	39.5 (10.0)
Non-Dutch origin, n (%)		15 (26)	25 (43)
**Education, n (%)**			
	No education or primary	16 (28)	16 (28)
	Vocational	18 (31)	24 (41)
	Secondary	9 (16)	9 (16)
	Higher	15 (26)	9 (16)
**DSM-IV^b^diagnosis**			
	Schizophrenia, n (%)	46 (79)	49 (85)
	Schizoaffective disorder, n (%)	1 (2)	5 (9)
	Delusional disorder, n (%)	1 (2)	0 (0)
	Psychotic disorder (not otherwise specified), n (%)	10 (17)	4 (7)
	Duration of illness in years, mean (SD)	13.3 (10.6)	14.9 (9.5)
**Medication use**			
	Antipsychotics, n (%)	54 (93)	57 (98)
	Olanzapine equivalent of prescribed antipsychotic medication (mg/day), mean (SD)	10.5 (6.8)	11.0 (8.3)
	Antidepressants, n (%)	15 (26)	17 (29)
Paid work, n (%)		8 (14)	5 (9)
Safety behaviors, mean (SD)		28.8 (14.2)	21.1 (16.0)

^a^VR-CBT: virtual reality-based cognitive behavioral therapy.

^b^DSM-IV: Diagnostic and Statistical Manual of Mental Disorders, Fourth Edition.

**Table 2:** Average per-participant costs per 3-month period in Euros for the year 2015 and average outcomes by measurement and condition.

Costs and outcomes		Baseline		Posttreatment (3 months)		Follow-up (6 months)	
		VR-CBT^a^	TAU^b^	VR-CBT	TAU	VR-CBT	TAU
**Costs (€), mean (SD)**							
	Health care costs	1918 (5178)	1396 (3146)	3031 (3189)	648 (960)	887 (1160)	1039 (2640)
	Travel costs	31 (23)	29 (26)	60 (34)	23 (15)	28 (22)	24 (16)
	Productivity loss	553 (2730)	224 (1214)	359 (1205)	214 (1127)	28 (161)	102 (588)
	Total (societal) costs	2502 (6246)	1649 (3570)	3076 (3469)	885 (1589)	943 (1185)	1165 (2766)
**Outcomes, mean (SD)**							
	GPTS^c^ paranoia (score)	85 (34)	77 (31)	70 (31)	75 (31)	67 (33)	75 (33)
	Time spent with others (proportion)	0.416 (0.256)	0.364 (0.266)	0.404 (0.226)	0.323 (0.266)	0.419 (0.209)	0.340 (0.273)
	Momentary anxiety (score^d^)	2.986 (1.120)	3.259 (1.484)	2.586 (1.089)	3.221 (1.495)	2.645 (1.095)	3.218 (1.388)
	Momentary paranoia (score^d^)	3.064 (1.393)	3.259 (1.418)	2.714 (1.291)	3.221 (1.518)	2.719 (1.293)	3.218 (1.467)

^a^VR-CBT: virtual reality-based cognitive behavioral therapy.

^b^TAU: treatment as usual.

^c^GPTS: Green’s Paranoid Thoughts Scale.

^d^Scores are on a 7-point Likert scale ranging from 1 (not at all) to 7 (very).

### Incremental Effects

#### Time Spent With Others

The treatment response rate regarding the time spent with others was 13 patients out of 58 (22%) in the control group and 24 patients out of 58 (41%) in the experimental group. The baseline-adjusted between-group difference between the response rates (ie, the incremental effect) was 0.23, which was statistically significant (SE=0.076, t_113_=3.07, 95% CI 0.08-0.38, *P*=.003).

#### Momentary Anxiety

The treatment response rate with regard to momentary anxiety was 17 patients out of 58 (29%) in the control group and 24 patients out of 58 (41%) in the experimental group. The between-group difference between the treatment response rates (ie, incremental effect) was 0.12, but this difference was not statistically significant (SE=0.089, t_114_=1.36, 95% CI –0.055 to 0.290, *P*=.18).

#### Momentary Paranoia

The treatment response rate in momentary GPTS paranoia was 11 patients out of 58 (19%) in the control group and 28 patients out of 58 (48%) in the experimental group. The between-group difference in the response rates was 0.29 and was statistically significant (SE=0.0841, t_114_=3.48, 95% CI 0.126-0.460, *P*=.001).

#### Quality-Adjusted Life Years

The SMD of GPTS paranoia was 0.523, which was statistically significant (SE=0.120, t_114_=4.37, 95% CI 0.285-0.760, *P*<.001). Using Sanderson et al’s conversion factor [14] of 0.1835 and taking into account a follow-up period of half a year, this became a QALY gain of 0.048 (0.523 × 0.1835 × 0.5) favoring the VR-CBT condition and this was statistically significant (SE=0.011, t_114_=4.37, 95% CI 0.026-0.069, *P*<.001).

### Incremental Costs

#### Incremental Health Care Costs

As can be seen in Table 2, the average per-patient health care costs in the TAU group was €1396 at baseline, €648 at posttreatment, and €1039 at follow-up. The average per-patient health care costs in the VR-CBT group was €1918 at baseline, €3031 at posttreatment, and €887 at follow-up. This includes €373.95 per patient for VR-related costs included in the posttreatment costs.

The cumulative costs per patient between baseline and follow-up, including the costs of VR-CBT, were €1686 and €3917 in the TAU and VR-CBT conditions, respectively. The between-group difference was €2231 (€3917–€1686) and was statistically significant (SE=663, t_114_=3.36, *P*=.001) when not adjusted for the initial cost difference between the conditions at baseline. After adjustment for baseline costs, the incremental health care costs became slightly less at €2170 and retained statistical significance (SE=661, t_113_=3.28, *P*=.001).

#### Incremental Costs Stemming From Productivity Losses

A total of 11.2% (13/116) of the participants had paid work. The average costs stemming from productivity losses per person for the TAU group was €224 at baseline, €214 at posttreatment, and €104 at follow-up. The average costs stemming from productivity losses per person for the VR-CBT group was €553 at baseline, €359 posttreatment and €28 at follow-up. The cumulative costs per patient between baseline and follow-up were €317 and €387 in the TAU and VR-CBT conditions, respectively. The between-group difference was €70 (€387–€317) and was not statistically significant (SE=274, t_114_=–0.26, *P*=.80).

#### Travel Costs

The average costs stemming from travel per person for the TAU group was €29 at baseline, €23 at posttreatment, and €24 at follow-up. The average travel costs per person for the VR-CBT group was €31 at baseline, €60 at posttreatment, and €28 at follow-up. The cumulative travel costs per patient between baseline and follow-up were €47 and €88 in the TAU and VR-CBT conditions, respectively. The between-group difference was €41 (€88–€47) and was statistically significant (SE=6, t_114_=–6.73, *P*<.001).

#### Incremental Costs From the Societal Perspective

The cumulative societal costs per patient between baseline and follow-up were €2050 and €4393 in the TAU and VR-CBT conditions, respectively. The between-group difference was €2343 (€4293–€2050) and was statistically significant (SE=747, t_114_=–3.14, *P*=.002).

#### Incremental Cost-Effectiveness Ratios From the Societal Perspective

The mean incremental costs for a positive treatment responder was as follows:

1. Time spent with others: €2343/0.23=€10,069.
2. Momentary anxiety: €2343/0.12=€19,525.
3. Momentary paranoia: €2343/0.29=€8079.
4. The mean incremental cost per QALY: €2343/0.048=€48,868.

**Figure 2:**
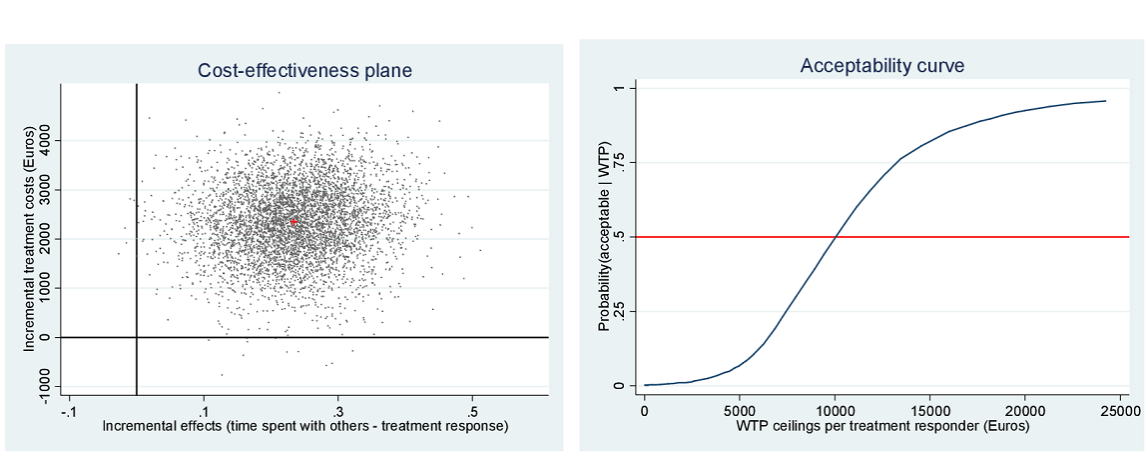
Cost-effectiveness plane and willingness to pay (WTP) acceptability curve for time spent with others.

**Figure 3:**
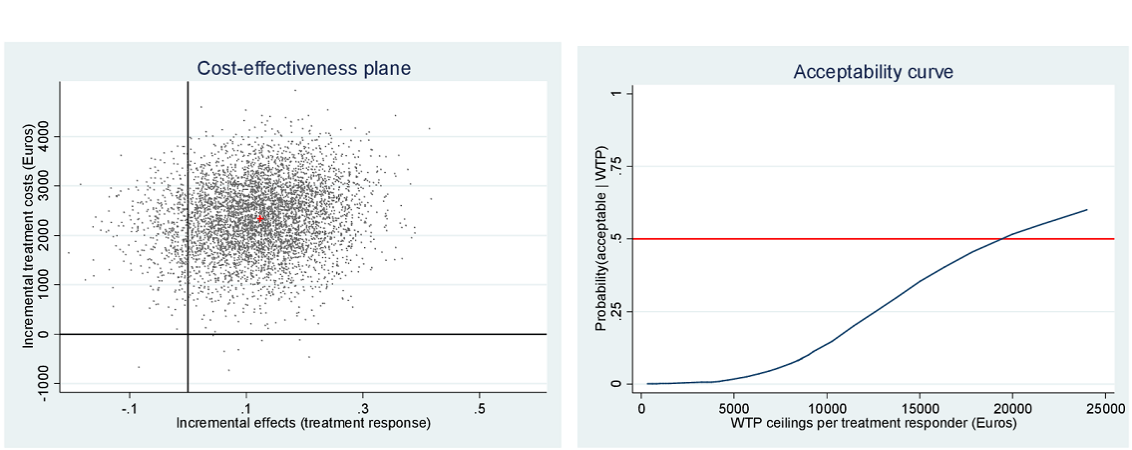
Cost-effectiveness plane and willingness to pay (WTP) acceptability curve for momentary anxiety.

**Figure 4:**
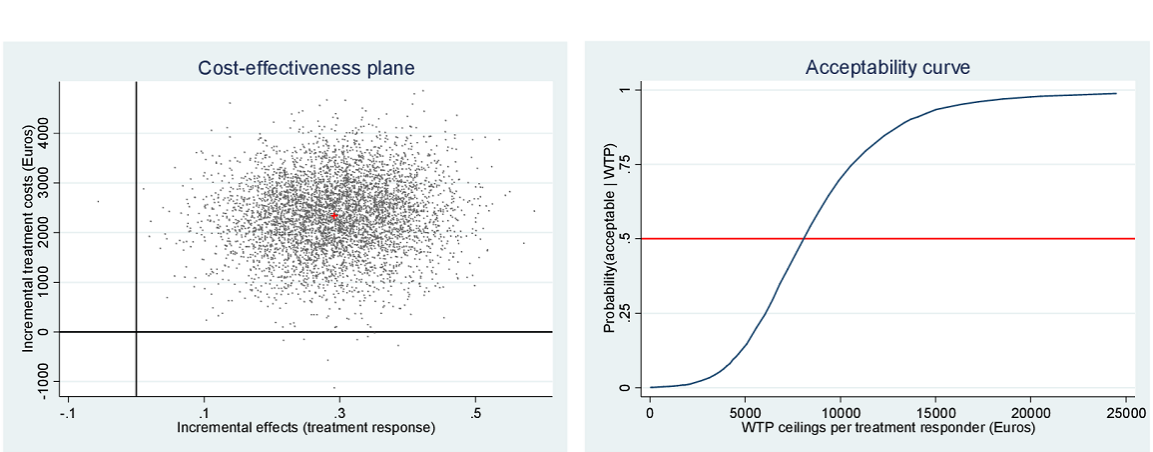
Cost-effectiveness plane and willingness to pay (WTP) acceptability curve for momentary paranoia.

Figures 2 to 4 depict the distribution of the 5000 bootstrapped ICERs over the cost-effectiveness plane for each of the social participation measures. Figure 2 depicts time spent with others, the plane illustrates 99.70% of the ICERs fall in the northeast quadrant, indicating that more QALYs are gained for higher costs. Figure 3 depicts momentary anxiety, the plane illustrates 90.74% of the ICERs fall in the northeast quadrant, indicating that more QALYs are gained for higher costs. Figure 4 depicts momentary paranoia, the plane illustrates 99.74% of the ICERs fall in the northeast quadrant, indicating that more QALYs are gained for higher costs.

**Figure 5:**
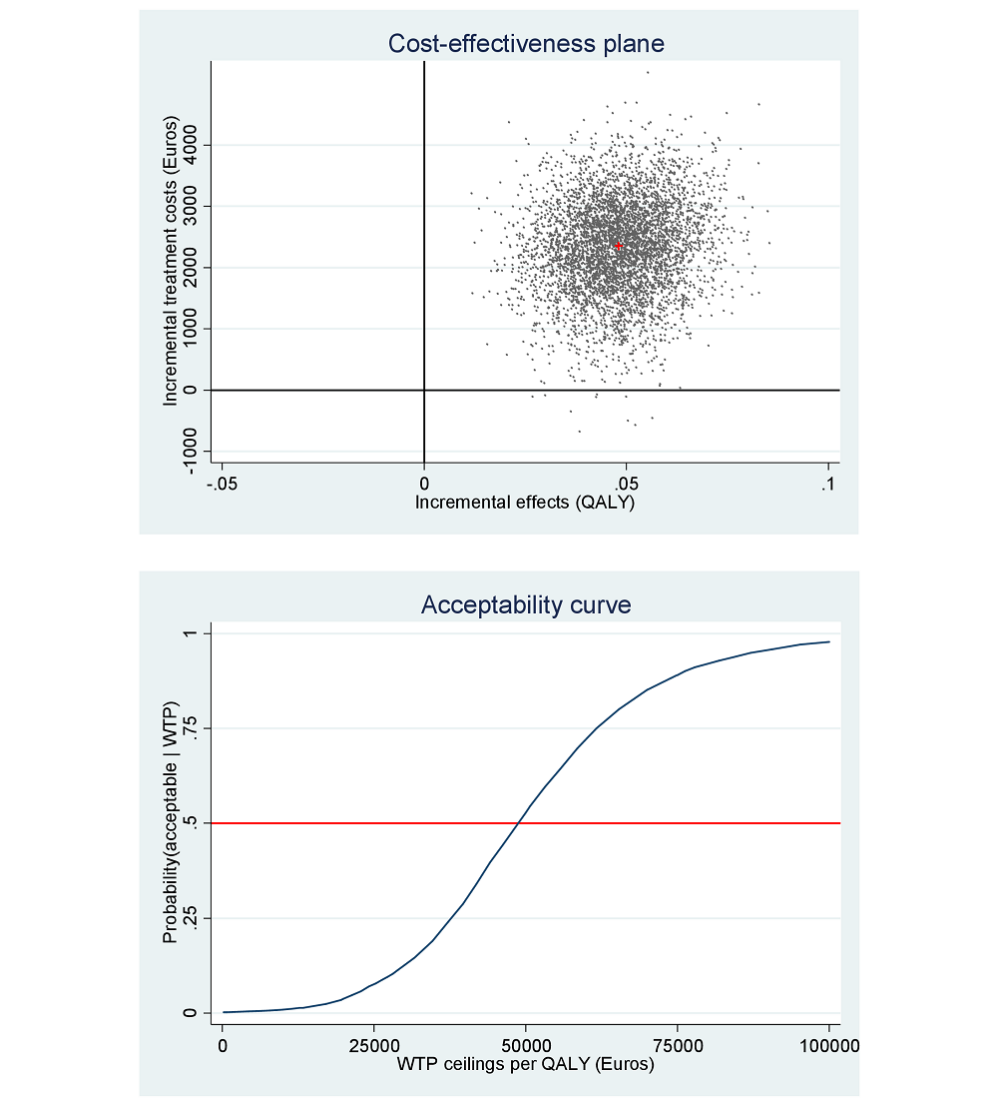
Cost-effectiveness plane and willingness to pay (WTP) acceptability curve for quality-adjusted life year (QALY) gain (costs per QALY gained) after 6 months.

Figure 5 depicts the distribution of the bootstrapped ICERs over the cost-effectiveness plane, with the vast majority of the ICERs in the northeast quadrant, indicating that more QALYs are gained but for higher costs, while 0.02% of the simulated ICERs fall in the southeast quadrant (ie, QALY gains for lower costs) for the VR-CBT group compared with the TAU group.

#### Acceptability

The mean incremental cost per QALY was €48,868. When looking at the acceptability curve in Figure 5, a higher probability that the VR-CBT intervention is deemed cost-effective can also be calculated. For an 80% certainty of cost-effectiveness, the incremental cost for gaining 1 QALY is €66,161, which falls well below the willingness-to-pay ceiling of €80,000 in the Netherlands for a severely disabling condition, such as schizophrenia characterized by paranoid delusions [21].

Looking at the three treatment responses, at 50% probability of being cost-effective, the costs are as mentioned: *time spent with others*, €10,069; *momentary anxiety*, €19,525; and *momentary paranoia*, €8079. Supposing that a decision maker needs an 80% certainty, *time spent with others* will have to be valued at €14,293 per treatment responder; *momentary anxiety* at €50,000; and *momentary paranoia* at €11,342.

#### Sensitivity Analysis

When including safety behavior at baseline as a covariate, the incremental costs per treatment responder on *time spent with others* became €9136; *momentary anxiety* became €17,535; and *momentary paranoia* became €7219. When including safety behavior at baseline as a covariate, the incremental costs per QALY gained became €44,597. Overall, incremental costs were somewhat lower when the baseline difference of safety behaviors was included in the analysis.

When including psychiatric admission costs at baseline as a covariate, the incremental costs per treatment responder on *time spent with others* became €9729; *momentary anxiety* became €18,879; and *momentary paranoia* became €7750. When including psychiatric admission at baseline as a covariate, the incremental costs per QALY gained became €47,308. Overall, incremental costs were somewhat lower when the baseline difference of psychiatric admission costs was included in the analysis.

When including both psychiatric admission costs at baseline and safety behavior at baseline as covariates, the incremental costs per treatment responder on *time spent with others* became €8592; *momentary anxiety* became €16,597; and *momentary paranoia* became €6800. When including both psychiatric admission costs and safety behavior at baseline as covariates, the incremental costs per QALY gained became €42,030. Overall, incremental costs were lower when the baseline differences of both safety behaviors and psychiatric admission costs were included in the analysis.

## Discussion

### Principal Findings

This study aimed to get an impression of short-term cost-effectiveness of VR-CBT for patients with paranoid delusions in comparison to TAU from a societal perspective. Data were collected 6 months after baseline at follow-up. Costs per treatment responder gained were estimated to be between €8079 and €19,525 for different aspects of social participation, with between 90.74% and 99.74% showing improvement. Cost per QALY gained at follow-up was estimated to be €48,868 with 99.98% showing improved QALYs. Sensitivity analyses showed costs to be lower when baseline differences in both safety behavior and psychiatric admission costs were included in the analysis. Costs per treatment responder gained were then estimated to be between €6800 and €16,597, with cost per QALY gained at €42,030.

### Results in Context

How much a society values solidarity with people burdened by disease will determine if guidelines are translated to actual treatment of patients. While the VR-CBT treatment condition is more expensive than TAU only, that was to be expected, as the aim was to add to existing treatment. Results show that this addition improves social participation for people with a psychotic disorder suffering from paranoid ideation. We see this improvement for time spent with others, momentary paranoia, momentary anxiety, and paranoid ideation, via the GPTS.

Engaging in psychological therapy is challenging for many patients suffering from paranoid ideation and treatment results vary. There are several aspects that favor VR treatment. Person-specific behavioral exposure is an important part of increasing treatment effect [22], which is exactly what the interactive VR social environments offer. Patients themselves also prefer VR over in vivo exposure treatment [23] and VR improves treatment motivation for patients [24].

Interestingly, during the follow-up we see that the VR-CBT group resulted in decreased health care costs and decreased costs due to productivity loss compared to the TAU-only group. There were no psychiatric admission days at follow-up for the VR-CBT group. To determine whether this was a coincidence or a trend, a much longer follow-up period is needed. Short-term societal costs were between €8079 and €19,525 for a positive treatment response. A disability weight of zero represents no loss of health and a weight of 1 represents health loss equivalent to death [25]. In the Netherlands, the willingness to pay for gaining a QALY ranges between €20,000 and €80,000 but differs per disease [26]. For a severely disabling disease such as schizophrenia, which according to the Global Burden of Disease study 2010 has a disability weight of 0.76, the willingness to pay is €80,000 [21,26]. In this context, the VR-CBT treatment that has an ICER of €48,868 per QALY gained can be regarded as acceptable from the cost-effectiveness point of view.

### Limitations

The study has several limitations. First, data were collected only 6 months postbaseline. Any longer-term effects and costs are unknown. There are indications that cost-effectiveness for treatment of psychotic symptoms improves with time [27] as health benefits continue. Second, minimal treatment response was set at a 20% symptom reduction. A 20% symptom reduction after just 8 weeks of therapy is clinically relevant in a patient group with an average duration of illness of 14 years with persistently high problematic isolation. Third, VR-CBT was compared to TAU only. The next step would be to compare VR-CBT directly to CBT, which is the current gold standard, as CBT without VR also results in additional costs to TAU. There are, however, also indications that VR-CBT could have positive results in fewer sessions compared to CBT [9]. Comparing VR-CBT directly to CBT also allows for the study of presumed benefits of VR therapy, such as better engagement and the ecological validity of VR on outcome effects. Such a study comparing VR-CBT and CBT on *time to response* and *costs* is currently ongoing (Netherlands Trial Register number NL7758). A final limitation was that QALYs were not measured directly. As the EQ-5D (European Quality of Life Five Dimension Scale) was not administered, QALYs were calculated using Sanderson et al’s conversion factor [14]. Future research needs to include the EQ-5D for direct measurement.

### Conclusions

This study found VR-CBT to be cost-effective in the short term from a societal perspective. However, the effect of additional VR-CBT sessions and long-term effects need to be determined while using direct measurement of QALYs.

## Appendix

Multimedia Appendix 1 Direct medical costs. Prices are from 2015 and are in Euros. *8 (sessions) × 2.5 (hours) × 2 (therapists) × 112 (Euros per contact-hour) / 8 (participants) = Є560. WRAP: Wellness Recovery Action Plan.

## Abbreviations

3DOF: 3 degrees of freedom
CBT: cognitive behavioral therapy
CEA: cost-effectiveness analysis
CEAC: cost-effectiveness acceptability curve
CHEERS: Consolidated Health Economic Evaluation Reporting Standards
CONSORT: Consolidated Standards of Reporting Trials
CUA: cost-utility analysis
DSM-IV: Diagnostic and Statistical Manual of Mental Disorders, Fourth Edition
EQ-5D: European Quality of Life Five Dimension Scale
ESM: ecological sampling method
GPTS: Green’s Paranoid Thoughts Scale
ICER: incremental cost-effectiveness ratio
ISRCTN: International Standard Randomised Controlled Trial Number
MICE: multiply imputed chained equations
QALY: quality-adjusted life year
SBQ-PD: Safety Behaviour Questionnaire-Persecutory Delusions
SMD: standard mean difference
SURE: seemingly unrelated regression equations
TAU: treatment as usual
TiC-P: Trimbos Institute and Institute of Medical Technology Assessment Questionnaire for Costs Associated with Psychiatric Illness
VR: virtual reality
VR-CBT: virtual reality-based cognitive behavioral therapy
VU University: Vrije Universiteit
