# Supplementary figures and images for: Cost-Effectiveness of Virtual Reality Cognitive Behavioral Therapy for Psychosis: Health-Economic Evaluation Within a Randomized Controlled Trial
Source: J Med Internet Res. 2020 May 5;22(5):e17098. doi: 10.2196/17098 (PMC7238085; doi:10.2196/17098)

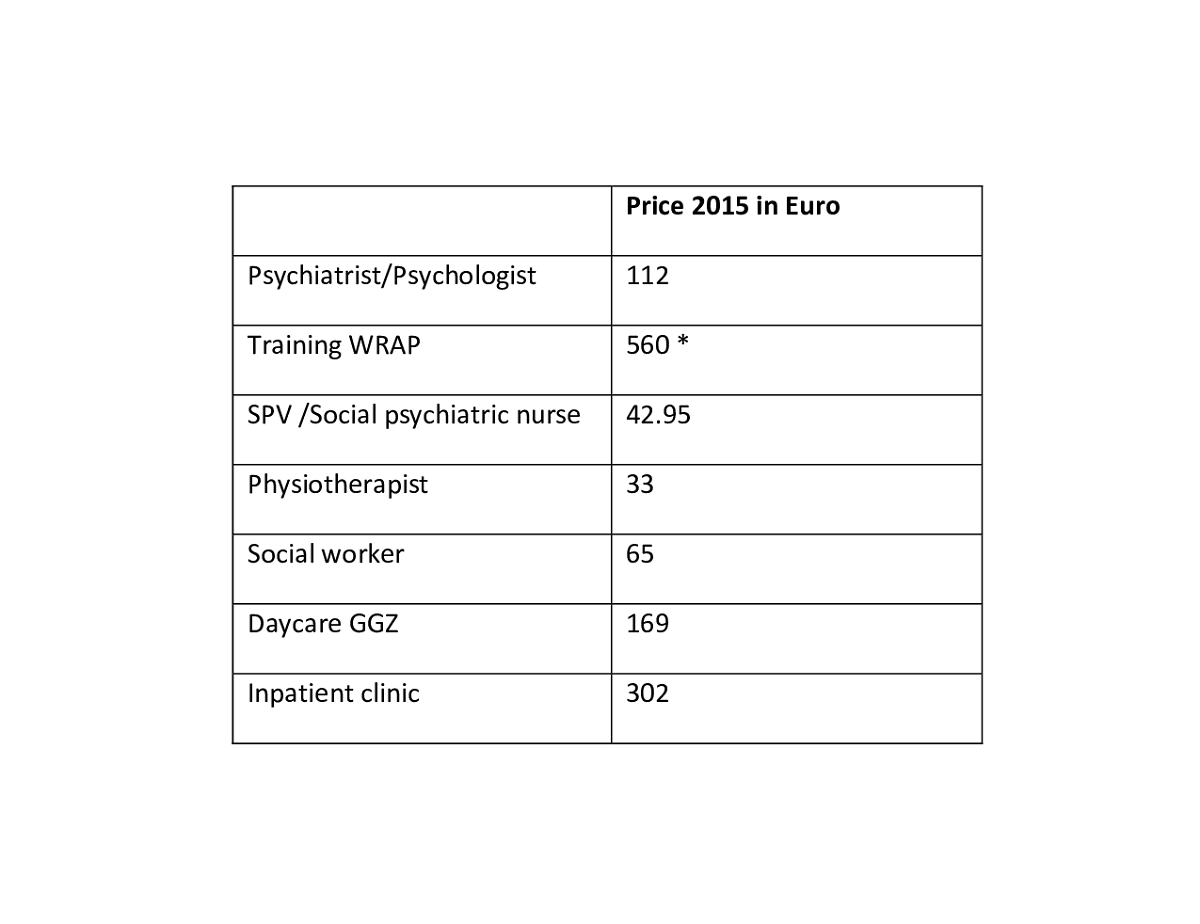

Supplement: Multimedia Appendix 1 [file jmir_v22i5e17098_app1.png]
